# Seeking balance between contradictory experiences - therapists treating survivors of sexual violence

**DOI:** 10.1080/17482631.2024.2422141

**Published:** 2024-11-02

**Authors:** Charlotta von Mentzer, Gun I Rembeck, Helena Dahlberg, Åsa Premberg

**Affiliations:** a Primary Health Care, Regional Health, Gothenburg, Sweden; b Institute of Health and Care Sciences, Sahlgrenska Academy, University of Gothenburg, Gothenburg, Sweden; c Primary Health Care Education, Research and Development, Research and Development Centre Södra Älvsborg, Region Västra Götaland, gothenburg, Sweden; d Borås Youth Guidance Center, Region Västra Götaland Regional Health, gothenburg, Sweden; e General Practice/Family Medicine, School of Public Health and Community Medicine, Institute of Medicine, Sahlgrenska Academy, University of Gothenburg, Gothenburg, Sweden; f Region Västra Götaland, Research and Development Centre, Primary Health Care, Gothenburg, Sweden

**Keywords:** Sexual trauma therapist, sexual violence survivor, secondary traumatization, qualitative research, thematic analysis

## Abstract

With the prevalence of sexual violence in most countries and its increase in Sweden, it is important to understand the development of secondary traumatic reactions among professionals who work with sexually violated clients. The aim of this study was to describe the meaning of therapists’ personal experiences when treating survivors of sexual violence. We conducted a qualitative interview study of therapists in Sweden (*N* = 11) using thematic analysis and adding a phenomenological openess towards the phenomenon. The participants were all women, with different professional backgrounds and with further education in areas such as psychotherapy, sexology, trauma treatment, and forensic nursing. The essential meaning of their work could be described as a continuum where therapists were seeking balance between contradictory experiences, further described in four themes. They experienced their work as highly meaningful, and the use of self-care strategies helped to maintain protective boundaries. However, the incomprehensible violence they were indirectly exposed to, challenged their protective boundaries, thus causing negative reactions for the therapists. Increased understanding of the impact of work on therapists’ professional and private lives is crucial, and the need for therapists to have a model or framework of meaning and explanation for sexual violence that ties contradictory experiences together.

It is documented that sexual violence—including sexual abuse, sexual assault, rape, sexual exploitation, and human trafficking for sexual purposes—presents a high risk of survivors developing post-traumatic stress disorder (PTSD) (Chivers-Wilson, 2006; Kessler et al., 2017; Ullman & Filipas, 2005). Research shows that therapists treating survivors of sexual violence, in turn, are more likely to develop secondary traumatization from exposure to the experiences of these clients than they are from exposure to traumas faced by other clients (Brady et al., 1999; Cunningham, 2003; Figley, 1995b; Johnson & Hunter, 1997; Kassam-Adams, 1999; Schauben & Frazier, 1995).

## Secondary traumatization

The phenomenon of secondary traumatization was first described by Figley (1983) as “the stress deriving from helping others who are suffering or who have been traumatized’ (Figley, 1999, p. 10). In the latest version of the Diagnostic and Statistical Manual of Mental Disorders (5th ed., DSM-5), secondary traumatization is included within the definition of PTSD: an individual must either have experienced a traumatic event themselves or have been exposed to aversive details of a traumatic situation (American Psychiatric Association APA, 2013).

Secondary traumatic stress (STS), compassion fatigue (CF), and vicarious traumatization (VT) are central terms used to describe the effects on therapists of exposure to painful material processed during therapy. STS includes symptoms such as hypervigilance, avoidance, depression, and exhaustion, which are identical to symptoms of PTSD (Gilbar et al., 2012; Salloum et al., 2015). CF is described as “a state of exhaustion and dysfunction, biologically, and emotionally” (Figley, 1995a, p. 253) and as the cumulative consequence of working with trauma survivors, enfolding feelings of confusion, isolation, helplessness, and symptoms of STS (Figley, 1995a). Pearlman and MacIan (1995) described VT as cumulative and permanent changes of a person’s cognitive schemas due to empathetic involvement with trauma survivors. Changes in a person’s cognitive schemas could mean, for example, that an earlier view of other people and the world as trustworthy could turn into a negative view of the world where “no one is trustworthy.” This in turn can lead to cynicism and depression.

There is a general acceptance in the literature that empathy and compassion play an important role in the development of secondary traumatization (Figley, 1995a; Rothschild, 2006; Sabo, 2011; Stamm, 2002) and that client recovery depends upon the professionals’ empathic ability, skill, and resilience (Figley, 1995a, 1995b; Pearlman & Saakvitne, 1995). However, the strong empathetic identification necessary to clients’ recovery increases the risk that caregivers could be negatively affected (Thomas & Wilson, 2004). Secondary traumatization can lead professionals to lose their ability to feel and express empathy and support for others and, consequently, it can have a negative impact on the results of therapeutic treatment (Hofmann, 2009).

Although STS, CF, and VT differ in some details, researchers sometimes use the terms interchangeably. However, “secondary traumatization” can be used as a generic term for the phenomena described above (Greinacher et al., 2019). Thus, in this paper we use the overall term “secondary traumatization” to describe all symptoms included in STS, CF, and VT, since there are no major differences between the concepts.

## Risk factors and resilience

Therapists with limited experience (Pearlman & MacIan, 1995) and those with more traumatized than non-traumatized clients are at higher risk of secondary traumatization than those with more experience and a more varied clientele (Schauben & Frazier, 1995). Excessive work demands (Maytum et al., 2004), too much responsibility (Melvin, 2012), and little or no knowledge about secondary traumatization are other known risk factors (Dominguez-Gomez & Rutledge, 2009; Meadors & Lamson, 2008; Melvin, 2012; Perry et al., 2011). Female therapists have been found to have a higher risk of secondary traumatization from working with traumatized clients than male therapists (Hooper et al., 2010; Kassam-Adams, 1999). Female therapists are also more likely than male therapists to report traumatic childhood experiences such as sexual abuse (Pope & Feldman-Summers, 1992), which are associated with a higher risk of secondary traumatization in work with traumatized clients (Cunningham, 2003; Kassam-Adams, 1999; Pearlman & MacIan, 1995). Some studies, however, show that therapists with a history of childhood sexual abuse do not appear to be at greater risk of secondary traumatization than therapists without such a history (Benatar, 2000; Schauben & Frazier, 1995; Simonds, 1996).

Knowledge and awareness of the symptoms and signs of secondary traumatization can help professionals avoid being negatively impacted by their work (Dominguez-Gomez & Rutledge, 2009; Meadors & Lamson, 2008; Melvin, 2012; Perry et al., 2011). Specialized supervision for trauma therapy, counselling (Pearlman & MacIan, 1995; Sodeke-Gregson et al., 2013), and peer support are protective against secondary traumatization. Safe and personal offices for therapists and regular meetings focused on secondary traumatization can also reduce the risk of negative effects (Pearlman & MacIan, 1995). Participating in stress-reducing and self-caring activities such as meditation, yoga, and other physical activities have also been shown to protect against secondary traumatization and to strengthen professionals’ resilience against negative impacts from work (Dominguez-Gomez & Rutledge, 2009).

Therapists can also be positively affected by their work with traumatized clients. Being able to help vulnerable people can contribute to quality of life, personal relationships, personal strength, and feelings of meaningfulness (Dyregrov & Mitchell, 1992; Pearlman & MacIan, 1995; Tedeschi & Calhoun, 2004; Yassen, 1995). Compassion satisfaction, the pleasure that comes from helping others, is a part of what motivates therapists (Stamm, 2002). However, sharing clients’ distress might also be an essential component of empathetic therapy, and therapists’ reactions and symptoms of secondary traumatization could be signs of their ability to process the clients’ trauma symptoms and enhance their post-traumatic growth (Gil, 2015).

With the prevalence of sexual violence in most countries and its increase in Sweden (BRÅ. The Swedish National Council for Crime Prevention, 2024; Svedin et al., 2021), it is important to understand the development of secondary traumatic reactions among professionals in the helping disciplines who work with sexually violated clients.

Although research has documented the impact of secondary traumatization on therapists and other professionals indirectly exposed to trauma, further work is needed to understand sexual trauma therapists’ perspectives on their experiences and challenges in therapeutic work with survivors of sexual violence. Accordingly, this study aimed to describe the meaning of therapists’ personal experiences when treating survivors of sexual violence.

## Method

Qualitative research can offer rich insights into the perspectives of health care professionals and patients on their experiences of health, illness, therapy, and care. The methods in this study are qualitative interviews and thematic analysis according to Braun and Clarke (2006, 2013). Qualitative individual interviews aimed to gain in-depth data and to reveal the multifaceted experiences of the participating therapists (Frances et al., 2013). Thematic analysis was first chosen to describe the personal experiences of therapists working with survivors of sexual violence because of its variability, flexibility, and usefulness in qualitative health care research. Thematic analysis can draw on different epistemological assumptions and can be used with different theoretical frameworks and methods that aims to search for, identify, and analyse patterns of meanings (Braun & Clarke, 2006, 2013). Recently, Braun and Clarke (2022) explained that thematic analysis is not a standardized method, and that they do not enforce strict adherence to the thematic analysis procedures. Their conclusion is that it’s important for the researcher to use their own theoretical, reflexive perspective and approach. In our study, one of our researchers has her expertise in phenomenological research as well as in phenomenological philosophy, and therefore we chose that the phenomenological approach was suitable as an epistemological foundation for the last part of the analysis. Openness is important in qualitative research and refers to the researchers’ previous knowledge and their way of approaching it in relation to their way of collecting and understanding data. Three of the authors are qualitative researchers and two of them have extensive experiences in qualitative methodology. All three are also midwives with broad clinical experience in various areas of the profession. These include working with young people in clinics dealing with sexuality and sexual health and in a gynaecological emergency clinic caring for women who have experienced sexual abuse and rape. The first author is also a trauma therapist with experience of working with survivors of sexual violence.

### Setting

Individual interviews were conducted with sexual trauma therapists working in five various units in Sweden that provide specialized treatment for survivors of sexual violence. These include public emergency units for survivors of sexual abuse and rape, public and non-governmental specialist clinics for non-emergency health care for adult survivors, and smaller units run by social services and youth clinics. Professionals working in the specialized units include midwives, social workers, and psychologists. Many of these professionals have further education in psychotherapy and trauma treatments such as eye movement desensitization and reprocessing (EMDR), prolonged exposure (PE), trauma-focused behavioural cognitive therapy (TF-CBT), somatic experience therapy, and life span integration therapy.

### Participants

We recruited a purposeful sample of therapists working with survivors of sexual violence through letters of information sent first by email to the heads of units in six clinics in various cities in Sweden providing specialized treatment for survivors of sexual violence. One unit turned down our request due to lack of time. After unit heads granted permission in the five remaining clinics, letters of information were sent to 11 therapists who had expressed interest in participating in the study. The therapists were all women aged 36 to 63 years. Their basic professions were midwife (*n* = 1), social worker (*n* = 8), psychologist (*n* = 1), and teacher (*n* = 1), and although not all were registered psychotherapists, they all had further education in areas such as psychotherapy, sexology, trauma treatment, and forensic nursing. Most participants had more than 8 years’ experience working with sexual trauma clients (experience ranged from 2.5 to 33 years), spent more than 50% of their working time with survivors of sexual violence, and met 10 or more sexual trauma clients per week. All offered individual treatments with clients, but five also offered group therapy.

### Data collection

Participants completed in-person interviews at a time and location convenient for them, which in all cases was at their offices. All interviews were conducted in Swedish by the first author from May to September 2018. Interviews were recorded and ranged from 33 to 61 minutes in length, averaging 43 minutes. The research question was broad and exploratory to elicit rich data about the therapists’ personal experiences: “Can you describe how you as a therapist experience therapeutic work with survivors of sexual violence?” Follow-up questions such as “Can you tell me more about that?” and “What do you mean by that?” were asked as well as questions about the impact of work on the therapists’ daily lives, their self-care strategies, and their personal experience of sexual abuse and rape.

### Data analysis

**Figure 1.**
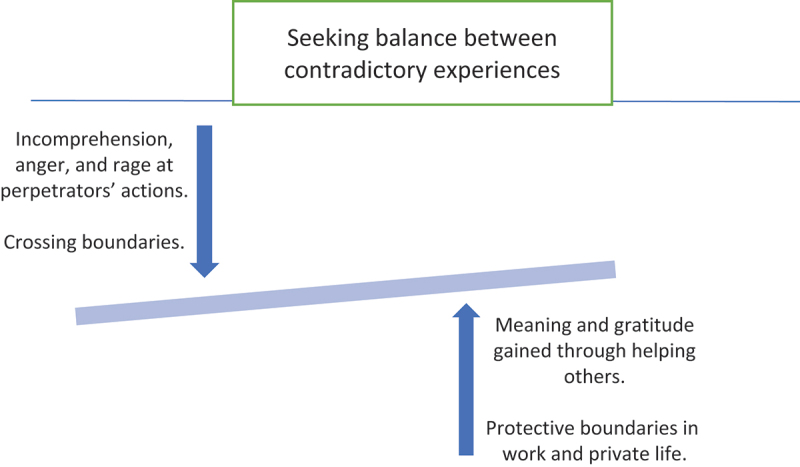
Thematic map of over-arching theme and subthemes.

In the first part of the analysis, the first and last authors cooperated in conducting the thematic analysis following Braun and Clarke’s (2006) six-phase guide. The interviews were transcribed verbatim and read and reread thoroughly. Initial codes were generated and collected into preliminary themes that were then reviewed, modified, and developed into defined themes using a thematic map. Quotations were selected, and the text was translated into English. In this part of the process, we identified a need for a further analysis to illuminate how the different themes that had been generated were related to each other and to determine whether there was an overarching theme holding all the meanings together. Braun and Clarke (2006) emphasize the flexibility of thematic analysis, stating that “thematic analysis is not wedded to any pre-existing theoretical framework and therefore it can be used within different theoretical frameworks” (p. 81) as well as combined with other methods. With this in mind, to deepen the analysis from the phenomenological idea to elucidate an essential meaning based on all data (Dahlberg & Dahlberg, 2019; Dahlberg et al., 2024; Dahlberg et al., 2008), we directed our analysis with the phenomenological openness and attentiveness towards the phenomenon in mind to find a theme that in an essential way offered new understanding of the phenomenon in focus. The demand for research openness here meant that we had to reflect on all levels of meaning, from the abstract, essential, and general to the more individual (Dahlberg & Dahlberg, 2019; Dahlberg et al., 2008). The original thematic map was then redefined to also include an overarching theme as well as candidate themes (see Figure 1).

### Ethical considerations

The study was conducted with approval from the Regional Ethical Review Board in Gothenburg, Sweden (Dnr: 156–18). The risk of discomfort in connection with participation in the interviews was considered. In this research project sensitive data relating to the participants’ experiences from therapeutic work with sexually traumatized clients and their personal experiences of sexual abuse was collected, which can mean that private and intimate spheres of people´s lives were highlighted, evoking thoughts and memories that could be difficult and stressful. This required a well-considered ethical approach from the start of the project to the publication of results.

## Findings

The essential meaning of working with sexually traumatized clients, which connects the four themes presented below, can be described as a continuum on which the participating therapists were moving in one direction or the other, seeking balance between contradictory experiences. While the therapists found their therapeutic work with survivors of sexual violence meaningful and they were gratified to be able to help others, they also felt incomprehension and rage at the perpetrators’ actions. They were aware of the importance of having boundaries and being strengthened by them, but they also found it difficult to maintain a protective distance between themselves and their clients and between their work and their private lives.

The therapists described being exposed to certain stories of torture and rape, dealing with young and very vulnerable individuals experiencing poverty and severe exposure, and dealing with cases of ongoing abuse and violence as some of the hardest aspects of their work. These aspects challenged the therapists’ professional shield and threatened to push them towards the negative pole of the continuum, causing physical, emotional, and mental reactions. Listening to their clients’ stories of the sexual violence they had endured was described by the therapists as entering an altered reality. They indicated that it was important to have an explanatory model to make the incomprehensible acts of sexual violence comprehensible, for their clients’ sakes as well as their own. The therapists also illuminated the importance of being aware of the impact of their work on both their professional and private lives, especially as women—in some cases, as women who had personally experienced sexual abuse.

The therapists’ contradictory experiences of therapeutic work with survivors of sexual violence are further described in four themes: Meaning and gratitude gained through helping others; Incomprehension, anger, and rage at perpetrators’ actions; Protective boundaries in work and private life; and Crossing boundaries. Quotes from the interviews are anonymized and identified by number as IP (interviewed person) 1–11.

### Meaning and gratitude gained through helping others

Therapists described their work with clients as highly meaningful. They thought being able to help others cope with having been sexually violated was both valuable and important, especially since they also thought such offences were generally neglected by society. The meaningfulness of their work was also connected with their feeling of making a difference and their overall satisfaction with their work. They were also grateful for their work because it benefitted them as well as their clients. They were constantly learning something new and were inspired by their clients’ courage. As one therapist stated, “I’m doing something in my life that feels very meaningful. It’s like I’m plugged into meaningfulness all the time” (IP 3). Another observed, “It gives something to that person, but at the same time it gives me something, too” (IP 11).

Helping others made the therapists feel happy and fulfilled at the end of the workday. They regarded their work as both fun and hopeful, since their efforts and treatments were often successful, even if it sometimes took years for that success to be recognizable. They saw results from their treatments in many ways, for example that the clients were able to have a healthy relationship with a partner or being able to start working or finish an education. The meaningfulness of their work also contributed to their feeling more emotionally available as human beings; working with survivors of sexual violence brought therapists closer to their own emotions and put them in touch with their own internal processes concerning for example existential questions of what is important in life. It also created feelings of gratitude and humility when they considered their own lives. One therapist said,

I think it is strange to say that you have a fun job working with the sexually traumatized, but it is a very nice and fun job. It’s also very positive to see that one can help and make a change. Even if it takes 1, 5, 7, or 10 years, it is possible to heal.

(IP 4)

Another therapist reflected on how her work affected her own outlook on life. “My work maybe affects me in the way that I’m more, even more caring, thankful, and humble for my own life and how well off I am” (IP 9).

### Incomprehension, anger, and rage at perpetrators’ actions

One of the hardest aspects of the therapists’ work was being indirectly exposed, when listening to clients’ stories of rape and abuse, to the incomprehensible violence, sadism, and torture committed by the perpetrators. Human-induced trauma like sexual violence, committed mainly by men towards women and children, were harder to comprehend and relate to compared to understanding and accepting traumatic events such as natural disasters or disease, and they awoke strong feelings of anger and rage in the therapists. As one therapist described it, “Sometimes it’s quite inconceivable, the things you hear, and you think, ‘Why are some people so terrible?’” (IP 7). Another said, “People being raped, that is a trauma caused by another human being. It can cause other feelings, especially rage, since it is another person doing something terrible to someone else” (IP 10).

Many of their clients recounted stories the therapists could not have imagined. Participants described feeling that, before working with sexually traumatized clients, they had been naive about what actually happens in society. They described feeling that these inconceivable stories seemed to come from another world, and that perception had a large impact on them. For example, “I felt sometimes when I finished work, what kind of world have I ended up in? And then when I came out of the office, well here is the normal world” (IP 2). The therapists indicated that it was very difficult to cope with their clients’ experiences of sexual trauma without having a logical explanation or model to make the incomprehensible comprehensible, both for their own sakes and for helping clients process the trauma. As one explained,

Many patients also ask, how could he do this to me? And then you need to be able to discuss that. You just can’t sit and be blank, you must believe something in those cases and try to help the patients find a model that is manageable and comprehensible. (IP 1)

### Protective boundaries in work and Private life

Therapists described being aware of the need to set protective boundaries between themselves and their clients, and between work and private life, as fundamental to their ability to cope with this work. They saw self-awareness—knowledge of one’s own strengths, limitations, and personal hardships—both as a person and as a therapist, as key to creating a professional distance between themselves and their clients, which they found were more necessary in their work with survivors of sexual violence than with other groups of clients.

I’ve always considered it very important during the years that I’ve worked with severely traumatized people to be careful not to project [their problems] onto myself or to get upset by thinking that anything about the client is really about me. (IP 6)

Therapists regarded the core of their therapeutic work was to help strengthening the client’s ability to create and maintain their own inner psychological boundaries, thus being in contact with and aware of their own needs and limits. Maintaining a professional margin could mean avoiding changes such as refurnishing the office, rescheduling appointments, or changing their personal appearance. The therapists also used self-care strategies to cope with lingering feelings of anger and fear after a session with a client, to help separating themselves from the clients’ experiences. Self-care strategies could include body scanning, dancing, yoga, and relaxation exercises.

If a client has been very scared and I have been infected by that, so to speak, and still feel fear in my body, I think it disappears in a way when I use self-care strategies like body scanning. I become more aware of my own boundaries: this is me; you are not in my space anymore. (IP 8)

They also considered it especially important to set boundaries between themselves and the clients when they had themselves experienced sexual abuse or rape, and they described it as impossible to cope with the work if their own experience was not processed and healed. The more similarities there were between their personal experiences and those of the clients, the more difficult it was to maintain those dividing lines.

If you have experienced sexual abuse yourself, I have understood that it can be complicated. Not impossible in any way, but I think it can be very complicated. If you have been in therapy and feel like you have processed it, then I think it can work. (IP 7)

There are so many incredible things that remind you, not just in the story, but also in how you react and deal with things, little things that you say or relate to that can also be a trigger for yourself. (IP 8)

It was also important for the therapists to clearly separate their work from their private lives to continue the work while still feeling good as individuals. It was important to be able to leave work behind at the end of the day and to have an active private life with physical activities and social contacts.

Something that I’ve learned is that when I go to work, I take off my private coat, and when I leave work, I take off my work coat. So, I don’t bring things home from work at the end of the day because then I wouldn’t feel so good. (IP 11)

Other ways to mark the difference between work and private life were to avoid reading or hearing about sexual abuse, exploitation, and rape during their time off and to surround themselves with decent men in their private lives. These strategies served to protect them from the inconceivable world of their clients’ abusive experiences and to emphasize the difference between their private lives and their work. Their therapeutic work with clients was about evil men doing harm to others, and such boundaries reassured them that their own private lives were not about that at all.

One therapist said that she protected herself by recognizing that not all men were abusers: “It’s important to surround yourself with good men. I have a husband, a son, and male friends who are good people” (IP 2).

The therapeutic work with survivors of sexual violence and their knowledge of the frequency of sexual abuse, rape, and exploitation in society also had an impact on therapists’ relationships with their children. They expressed the need to set protective boundaries against the risk of their children becoming either victims or perpetrators of sexual abuse or rape. Their knowledge of where sexual abuse and rape commonly occur, for example, made them reluctant to allow their children to sleep over at a friend’s home if they did not know the friend’s parents. Being sensitive to their children’s own limits, teaching their children to say no and to object to things they might be uncomfortable with, and raising their sons to treat all genders with respect were various ways in which therapists tried to protect their own children, as illustrated by the following statements: “I’m more aware of all the risks, where abuse happens, and I have that with me in a totally different way than I see other parents have” (IP 8); “I think that if I hadn’t been working here, then I maybe wouldn’t have raised my sons in the way I’m doing now. Now I am very clear about boundaries” (IP 4).

Support from the closest colleagues was described as a safety factor in work as well as having professional supervision. All the therapists were provided supervision through their work but to a various degree. To do this kind of work without supervision was regarded as almost impossible and unacceptable, as one of the participants described it: “I don´t work without supervision. Absolutely not” (IP 6).

### Crossing boundaries

Therapists described being exposed to certain aspects of clients’ experiences, history, or situation as challenges to their protective boundaries that caused physical, emotional, and mental reactions. Very vulnerable individuals, those who were very young, living in poverty, experiencing severe exposure, or who had been abandoned were some of the most difficult for therapists to cope with.

I’m affected a great deal by a person’s vulnerability. I know there are certain things I find harder, for sure. I don’t think terrible stories per se are more difficult, but if they contain a lot of loneliness, then I can become overwhelmed by it. (IP 8)

They also described how much more difficult it was to deal with cases of ongoing sexual violence than with sexual trauma from the past. Ongoing sexual violence awoke protective instincts within the therapists but also made them wish to end the therapy because it was too hard to cope with.

I find it easier to hear terrible stories about things that have happened [in the past], but in certain cases it has emerged that it’s still happening, and then it has been very difficult. You end up in some kind of situation where you both want to continue and at the same time you can’t work with this, because it’s not possible. You feel protective and do not want to abandon [them], but at the same time you want to quit. (1P 5)

Sometimes therapists felt that their clients’ situations or stories crossed over into their own private spheres. For example, clients whose childhood sexual abuse happened when they were at about the age of the therapists’ own children was experienced as coming too close to the therapists’ own lives.

Coming home after you’ve heard a story about how a three-year-old was sexually abused and then putting your own three-year-old to bed at night—that was a little bit too much, a little bit too much to bear, because it was too close to my own child. (IP 4)

As another example, a client who was being sexually abused by a man who was stalking her roused worries in the therapist that the perpetrator could come after her as well.

It’s some kind of damned mafioso who just collects her and uses her, and he can be anywhere and just show up with his van, and he drags her in, and he seems to know exactly where she is. (IP 5)

In some situations, therapists felt the crossing as a physical reaction during therapy. Exposure to certain stories could be overwhelming, and the therapists sometimes felt as if the feelings and experiences of the clients passed straight through into them. This happened particularly when the therapist was unprepared; certain details and stories could surprise them and pass through the professional shield they had tried to build up through their working experience. Physical reactions such as tunnel vision, body tension, losing one’s voice and ability to speak, and seeing persistent and disturbing inner visions were some of the reactions the therapists experienced as they felt invaded and overwhelmed by the clients’ stories and experiences. For example: “I feel it in my body. It doesn’t go up to my brain, but it goes straight in and then I feel my body getting all tense” (IP 2); “Sometimes, some things they tell you can just pass through that shield that you have and then turn into a picture or something that is hard to get rid of” (IP 1).

The consequences of having their boundaries crossed included difficulty letting go of work at the end of the day, having nightmares, and flashbacks: “I couldn’t let go of work when I came home. I dreamed of work at night and had nightmares” (IP 4). Some therapists also found themselves feeling stressed, afraid, and suspicious of other people. These reactions made it difficult to cope with work and could lead to sick leave, increased need of professional supervision, and an inability to work with certain types of clients for a period of time:

I was very stressed; I had flashbacks quite a lot. I started to look around at people and I felt no trust toward anyone. I felt like everyone came toward me in a creepy way and I had nightmares and couldn’t sleep. (IP 8)

Being overwhelmed and exposed to too many traumatic stories over too long a time also caused mental fatigue and drained therapists of the energy needed to socialize outside of work. In this situation, therapists would lose interest in social gatherings, from casual conversations to larger get-togethers and parties, finding them boring and meaningless. The therapists described having difficulties in their relationships with other people because they lacked the strength to let new people in and to listen to their stories. They also expressed feeling mentally “heavier” and generally in a lower mood after working for an extended time with sexually traumatized clients: “Well, I think that it makes you heavier, like your mood steps down a level over time” (IP 3). They also had less patience with people who became upset about what the therapists thought were petty concerns, and they were more likely to lose their patience with their own children and even certain clients:

I hardly have the energy to have any kind of private life during the semesters. Well, you don’t have the energy to meet people and there are many colleagues who say the same thing when their phone rings. “Oh no, do I have to speak to somebody?” And that’s the price you must pay. (IP 1)

Spending time alone was deemed necessary to being able to wind down and feel calm after work. The insight that their private social network had shrunk and their perceived need to be alone had increased came gradually to the therapists; they tended not to notice it until after some years of working in sexual trauma therapy and counselling. Becoming a much less social person was seen as a consequence of working in sexual trauma therapy and the price they had to pay. However, the extent to which these changes affected their work, both as professionals and as women—some with personal experiences of sexual abuse and rape—seemed not to be fully understood or taken seriously, neither by the therapists themselves nor by their colleagues and bosses. “Not trivialize but not quite understand or take seriously how it affects us to sit and hear these stories. Also being a woman with my own experience” (IP 2).

## Discussion

The essential meaning of working with sexually traumatized clients can be described as a continuum on which the participating therapists were moving in one direction or the other, seeking balance between contradictory experiences. On one side, there were feelings of meaningfulness and gratitude, and protective boundaries were maintained, and on the other, feelings of rage and incomprehension, and of having one’s protection crossed. Some of the hardest aspects for the therapists in this study to handle in their work were the incomprehensible violence and atrocities they were indirectly exposed to, as well as facing cases of ongoing sexual violence and dealing with young and very vulnerable clients. These aspects threatened to push the therapists towards the negative pole of the continuum and cause negative reactions. Because dealing with cases of ongoing sexual violence and with vulnerable and young clients may awaken particularly high levels of empathy, these aspects may also increase professionals’ risk of being vulnerable to stressors causing physical, emotional, and mental reactions (Dombo & Gray, 2013; Figley, 1995a; Rothschild, 2006; Sabo, 2011; Stamm, 2002; Thomas & Wilson, 2004). A recent Swedish study exploring the psychological effects and potential difficulties medical personnel and police officers experience in their meetings with women who have been raped also described participants having more empathetic involvement with survivors from socially vulnerable groups, such as those with addiction and those experiencing homelessness (Rudolfsson & Sinani, 2022).

The participating therapists in our study all acknowledged that it was necessary for them to maintain borders in the therapeutic situation but also in private life, for example, concerning their own children, and they described different self-care strategies they used. The therapists also expressed a need to surround themselves with good men in their private lives in contrast to the harmful men who were a reality for their clients. They described these protective boundaries around their families and themselves as resulting from the fact that they were more aware and knowledgeable than most people about the frequency of sexual violence in society. Earlier research on female medical personnel meeting sexually abused women in Sweden described the medical professionals becoming more suspicious of men, viewing most men as potential rapists, and even feeling hatred towards most men as a result of their increased knowledge and awareness of men’s violence against women in society (Rudolfsson & Sinani, 2022).

Despite the participating therapists’ efforts to set functional dividing lines, they all reported contradictory experiences of those lines being crossed when they were exposed to clients’ vulnerability and the atrocities they had suffered. The therapists described various reactions, such as invasive imagery, nightmares, hypervigilance, distrust of others, mental fatigue, and social withdrawal as inevitable consequences of being overwhelmed. The therapists’ reactive avoidance of social events and contact with others and their increased need for time alone that became apparent in this study contrasts with the finding that they also acknowledged needing a social life to feel well. This contradictory finding might point towards a risk that secondary reactions due to crossed boundaries can gradually erode therapists’ ability to continue self-care strategies and thereby further reinforce negative reactions and weaken their resilience.

The importance of professionals establishing and maintaining protective strategies in work with traumatized clients to alleviate the risk of secondary traumatization that became apparent in this study echoes earlier research where friends and family were important in helping professionals separate their work from their private lives, and self-care strategies helped to strengthen interpersonal boundaries (Harrison & Westwood, 2009; Hunt, 2018). Our finding that the protective strategies extended to the therapists’ relationships with their own children is consistent with earlier findings on the experiences of female employees in rape crisis centres, who also showed increased protectiveness of their own children (Clemans, 2004).

The participating therapists in our study described how they failed to comprehend or find meaning in their clients’ stories of violence and abuse, creating strong feelings of anger and rage at perpetrators’ actions. They experienced incomprehension and confusion as if entering an alien world or altered reality when hearing their clients’ stories of rape and abuse, and they found it especially difficult to comprehend sexual and interpersonal trauma since such trauma is caused by another human being, unlike trauma caused by disease or natural disaster. It was found to be important for therapists to have some kind of explanatory model to present to their clients—and also to aid their own understanding—as to why these terrible things happen and why some men can commit such actions.

Secondary traumatization can cause professionals to have invasive and disturbing cognitions of their clients’ traumatic experiences, which can alter their own perspectives on the world and themselves, causing feelings of incomprehension and confusion (McCann & Pearlman, 1990; Neumann & Gamble, 1995), as was described by the therapists in this study. Park’s (2010) integrated meaning-making model holds that distress ensues when our assumptions about our lives, ourselves, and the world are contradicted by a stressful life event or adversity. This model might clarify the therapists’ experiences of disbelief and disorientation when exposed to stories of sexual violence in their therapy sessions with clients. Professionals are often unaware and uninformed about how working with traumatized clients can radically change their view of the world and of others (Wilson, 2016). Educational and training programmes for professionals who work with traumatized and abused clients often lack sufficient focus on comprehensive understanding of violence and trauma and on preparing them for secondary traumatization. This lack of knowledge and preparation can lead to therapists’ inability to develop strategies to prepare and protect themselves from the negative effects of their work (Howlett & Collins, 2014).

The therapists in this study struggled to maintain protective boundaries and thereby prevent negative aspects of their work from entering their personal and private lives. However, setting boundaries could also be seen as a defective coping mechanism, in which symptoms such as hypervigilance and increased distrust of others makes the boundaries function more as emotional barriers or buffers (Neswald-Potter & Simmons, 2016). According to existential therapy, disturbance and health are two sides of the same coin as well as good and bad, and we all need to see both in our lives. The well-being of sexual trauma therapists coincides in this view with the therapists’ ability to be transparent and open to the understanding that life can bring both good and bad and hence can include experiences of sexual violence (van Deurzen et al., 2019). Being open and transparent and at the same time having boundaries or buffers for protection can seem like a difficult challenge to manage for professionals working with sexual trauma. The participating therapists’ descriptions of feeling like they were entering a different world or altered reality when exposed to clients’ stories of atrocities and sexual violence might be seen as examples of the difficulties that many people might share in comprehending or seeing certain horrific and frightening aspects of life as part of reality. Hence the need for barriers to keep this altered or other reality outside our own.

There are still a lot of taboos, stigmas, and feelings of shame connected to sexual violence that influence our ability to talk about experiences of sexual trauma (Deitz et al., 2015; DiMauro & Renshaw, 2021; Rajan et al., 2021). To some extent, these might also affect professionals working with the survivors. Descriptions such as *unmentionable actions* or *unspeakable crimes* show how our human perspective of the world is related to the language. To be able to see and to comprehend things, we need to name them and be able to talk about them. If we lack words or concepts or the ability to speak, it might be an obstacle to developing explanatory models for aspects of life that are considered inconceivable.

The question is, what do professionals need to be able to create explanatory models of meaning that tie contradictory experiences together, hence making the incomprehensible aspects more comprehensible in their work with survivors of sexual violence? Religion and personal faith played a vital role for maintaining well-being in humanitarian professionals exposed to cybercrime child sexual exploitation in the Philippines, according to a study by McCormack and Lowe (2022). In the study, the participants’ personal faith served as a framework by which experiences could be understood, and it offered support for longevity and sustainability in demanding work. For those professionals without a personal faith or connection to a religious belief they could use as a model of meaning and explanation, further research is required in order to support them in their work treating sexual trauma survivors. Professional supervision, organizational structure, and increased knowledge about the impacts of their work on both their professional and private lives are very important factors contributing to therapists’ ability to cope with their work. The breaking of taboos and stigmas surrounding sexual violence could possibly be helpful in finding words and concepts that unveil certain incomprehensible as well as unmentionable aspects in the real world. In this way there would be a red thread between naming and seeing and then acting. A framework or model of meaning and explanation that ties contradictory experiences of working with sexual trauma together might be a possible result of this red thread.

## Limitations

Qualitative research results must always be understood in their own context and should never be interpreted as universal. This caveat, however, does not exclude the possibility that the results might be understood outside the immediate context (Kvale, 2001). Although this study was undertaken in a Swedish context with female therapists working with survivors of sexual violence, it might also inform therapeutic work with sexual trauma clients in other contexts. The purposeful sample of sexual trauma therapists in this study could be regarded as reasonably characteristic in that most sexual trauma therapists in Sweden are female, and the participating therapists had long working experience and wide-ranging insights and knowledge about sexual trauma. Their ages varied, and most of the units in Sweden providing specialized treatment for survivors of sexual violence were included.

The interviews were conducted in 2018, which might be a limitation considering the time that has taken place until the publication of the results. The aim of the study was to describe the meaning of the therapists’ personal experiences when treating survivors of sexual violence. There is an increase in self-reported sexual violence in Sweden (BRÅ. The Swedish National Council for Crime Prevention, 2024; Svedin et al., 2021) since the interviews were conducted thus possibly contributing to an even bigger need for therapeutic treatments and interventions and an even higher relevance for further investigating professionals’ experiences of working with sexually traumatized clients.

The first author’s experience as a sexual trauma therapist herself could be seen as a limitation possibly affecting the result. However, authors practiced openness and were open about their pre-understandings, and such self-awareness, together with a reflective attitude, has been an important consideration throughout the study.

## Conclusion

According to the findings in this study, working with sexually traumatized clients could be described as a continuum on which the participating therapists were constantly moving in one direction or the other, seeking balance between contradictory experiences. On one side, they experienced their work as highly meaningful, and they used different self-care strategies to maintain protective boundaries around themselves at work and in private life. On the other side, the incomprehensible violencethey were indirectly exposed to when listening to clients’ stories of sexual violence, as well as dealing with young and very vulnerable clients and clients facing ongoing abuse, were described by the therapists as some of the hardest aspects for them to handle, challenging their protective boundaries and causing negative reactions for the therapists. Findings from this study show that increased understanding of the impact of work on therapists’ professional and private lives is crucial, and that there is a need for therapists to have models of meaning and explanation for sexual violence. A model or framework of meaning and explanation that ties contradictory experiences together could possibly contribute to creating increased balance and sustainable working conditions for those who care for the survivors.
